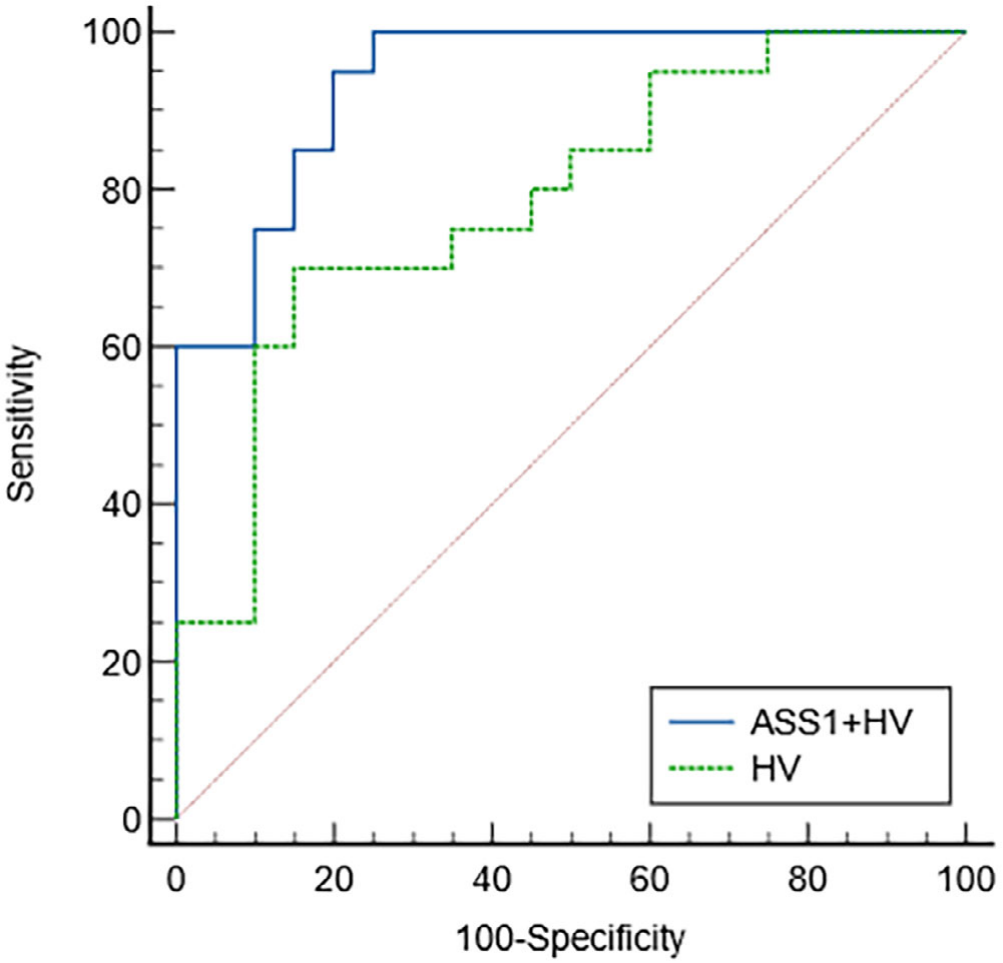# Application value of structural MRI combined with ASS1 in early diagnosis of Mild Cognitive Impairment

**DOI:** 10.1002/alz.089140

**Published:** 2025-01-09

**Authors:** Lele Chen, Xiang Fan, Hui Shan, Keyan Yu, Gaigai Lu, Zhuonan Wei, Guanxun Cheng

**Affiliations:** ^1^ Peking University Shenzhen Hospital, Shenzhen, Guangdong China

## Abstract

**Background:**

Mild cognitive impairment (MCI) refers to a transitional stage between the cognitively unimpaired (CU) and dementia, which is probable in the Alzheimer's disease (AD) continuum. Neuropsychological assessments and structural MRI are primary clinical examinations applied for diagnosing MCI. Hippocampal volume (HV) and medial temporal lobe atrophy (MTA) as recognized AD biomarkers are the most frequently used imaging markers in clinical application. However, HV or MTA is not sensitive enough in the early stage of AD. Recently, blood‐based biomarker analysis has demonstrated great potential for early AD diagnosis because it is simple and less invasive. It has been shown that argininosuccinate synthetase (ASS1) expression is increased in Alzheimer's disease neurons and glial cells, which also correlates with inducible nitric oxide synthase (iNOS) expression, and that there is a link between the increased NO production observed in Alzheimer's disease brains and iNOS expression. We aim to study whether ASS1 can provide additional value compared with HV in diagnosing MCI and validate the diagnostic utility of the combined model.

**Method:**

Twenty MCI patients and 20 CU were recruited and underwent neuropsychological assessment, MRI examinations, and the collection of blood. Protein quantification was analyzed by high‐resolution tandem mass spectrometry (MS/MS), and the ROC curve was plotted for selected biomarkers. The automated structure tool and SPSS 26.0 were used to analyze the HV and ASS1. We investigated the area under curve (AUC) of HV, and the combined model using both ASS1 and HV based on the logistic regression. The Delong test is used to compare the combined model with the single HV.

**Result:**

The AUC of diagnosing MCI by HV was 0.788 (95%CI: 0.629 ‐0.901). When combining HV with ASS1, the AUC increased to 0.938 (95%CI: 0.813‐0.989). The Delong test results indicated a significant difference between the single HV and the combined model. (P=0.0188).

**Conclusion:**

Combining plasma ASS1 could provide significantly additional value in detection of MCI, which also means plasma ASS1 and HV may be a potential early biomarker combination in diagnosing early AD.